# Podocyte specific exon skipping after disease onset improves kidney pathology and function in a mouse model of Alport syndrome

**DOI:** 10.1038/s41598-025-25447-w

**Published:** 2025-11-25

**Authors:** Kentarou Hashikami, Ryosuke Kobayashi, Ryotaro Hori, Kenta Danbayashi, Yasunori Nio

**Affiliations:** 1 Pharmacology Business Unit, Metabolic Syndrome Group, Axcelead Drug Discovery Partners, Inc., 26-1, Muraoka-Higashi 2-Chome, Fujisawa, Kanagawa Japan; 2 Pharmacology Business Unit, Genetically Modified Animal Group, Axcelead Drug Discovery Partners, Inc., Japan 26-1 Muraoka-Higashi 2-chome, Fujisawa, Kanagawa,; 3 Pharmacology Business Unit, Integrated Pathology Group, Axcelead Drug Discovery Partners, Inc., 26-1 Muraoka-Higashi 2-chome, Fujisawa, Kanagawa, Japan

**Keywords:** Alport syndrome, Post disease onset, Exon skipping, Podocyte, UACR, Collagen 4a5, Diseases, Drug discovery, Genetics, Molecular medicine, Nephrology

## Abstract

Alport syndrome (AS) is a hereditary kidney disorder caused by mutations in *COL4A3*, *COL4A4*, and *COL4A5*, which often lead to progressive renal failure. Although angiotensin II receptor blockers are available for symptomatic treatment, no radical or curative therapies currently exist. Given the genetic basis of AS, exon-skipping therapy has the potential to serve as a definitive treatment. In this study, we established a tamoxifen-inducible exon 21–skipping mouse model, enabling the evaluation of post-onset therapeutic intervention. We generated a novel AS mouse model harboring a patient-derived nonsense mutation (R471*) in exon 21 of *Col4a5* on a C57BL/6 background. Using this strain, we further developed a tamoxifen-inducible, podocyte-specific exon 21–skipping model. Induction of exon skipping, either before or after disease onset, restored truncated collagen IV α5 expression, improved renal function, and ameliorated glomerular and tubular pathology. Notably, treatment initiated after the onset of proteinuria reversed glomerular injury, underscoring its therapeutic potential even in progressive stages. This model provides a robust platform for evaluating mutation-targeted therapies and supports the feasibility of exon-skipping approaches for AS and other monogenic kidney diseases.

**Supplementary Information:**

The online version contains supplementary material available at 10.1038/s41598-025-25447-w.

## Introduction

Alport syndrome (AS) is an inherited form of chronic kidney disease (CKD) characterized by progressive glomerular damage that ultimately leads to kidney failure. Its prevalence is estimated to range from 1 in 2,000 to 1 in 53,000 individuals, depending on diagnostic criteria and the extent of genetic screening^1–3^. The pathogenesis of AS is rooted in structural abnormalities of the glomerular basement membrane (GBM), which are predominantly caused by mutations in the type IV collagen genes—*COL4A3*, *COL4A4*, and *COL4A5*^4–7^. These genes encode the α3, α4, and α5 chains of type IV collagen, which are integral components of the mature GBM.

Deficiencies or dysfunction in these collagen chains compromise GBM integrity and impair its filtration capacity, leading to hematuria, proteinuria, glomerulosclerosis, and tubulointerstitial fibrosis accompanied by inflammatory infiltration^8^. Emerging evidence indicates that AS progression shares pathological mechanisms with other CKDs, including inflammation, fibrosis, oxidative stress, and mitochondrial dysfunction^9^ A notable large-scale whole exome sequencing study has revealed that mutations in *COL4A3*, *COL4A4*, and *COL4A5* collectively account for nearly 30% of all monogenic CKD cases, establishing AS as the second most common hereditary nephropathy after autosomal dominant polycystic kidney disease^10^.

This underscores the broader clinical relevance of AS—not only as a rare disease but also as a significant contributor to the overall burden of hereditary renal disorders. Unlike most CKD cases, which are associated with hypertension, diabetes, or vascular disease, AS is a genetically driven nephropathy that occurs independently of these common risk factors. Approximately 80% of AS cases are X-linked (XLAS), resulting from *COL4A5* mutations, with the remainder exhibiting autosomal recessive or dominant inheritance due to *COL4A3* or *COL4A4* mutations^11–13^.

Currently, there is no curative treatment for AS. The most widely adopted therapeutic intervention involves angiotensin II receptor blockers (ARBs), such as irbesartan, which mitigate disease progression by reducing intraglomerular pressure and proteinuria^14^ However, ARBs are largely palliative, offering limited efficacy in preventing or reversing the intrinsic structural damage characteristic of AS.

Given the high likelihood of progression to end-stage kidney disease and the lifelong burden of the disease, the development of more effective disease-modifying treatments remains an urgent clinical need. Gene therapy approaches using viral vectors, such as adeno-associated viruses or retrovirus-based systems, have been explored in preclinical models. Nevertheless, these methods face substantial hurdles, including inefficient renal delivery, immunogenicity, and off-target effects^15,16^ In this context, antisense oligonucleotide (ASO)-mediated exon-skipping therapy has emerged as a promising and targeted approach. Nozu et al. have demonstrated that exon 21 skipping in the *Col4a5* gene using ASOs could circumvent a premature termination codon (R471*), resulting in an in-frame deletion and the expression of a truncated yet partially functional α5(IV) collagen chain^17^.

This intervention significantly improved renal pathology and clinical parameters, such as urinary albumin/creatinine ratio (UACR), serum creatinine, and blood urea nitrogen (BUN), ultimately prolonging survival in a previously established humanized AS mouse model^18^ Mechanistically, ASOs were designed to target exonic splicing enhancers or splice junctions, thereby modifying pre-mRNA splicing to exclude the mutant exon while preserving the reading frame^19^ This enables the production of a semi-functional protein and mitigates the deleterious effects of the premature termination codon.

Despite these promising results, previous studies have primarily used prophylactic ASO administration before overt disease onset. For translational relevance, it is imperative to determine whether therapeutic ASO delivery after the disease onset has renoprotective effects. To address this gap, we established a tamoxifen-inducible exon 21 skipping mouse model that enables temporal control over ASO activation. This design allowed us to investigate the therapeutic efficacy of post-onset exon skipping in restoring COL4A5 expression and ameliorating the disease phenotypes. Importantly, this model serves as a robust platform not only for evaluating ASOs but also for testing mRNA supplementation and gene-editing strategies in the context of established renal pathology.

Our findings provide key insights into the optimal timing, therapeutic potential, and clinical translation of exon-skipping therapy for AS, thereby advancing the development of meaningful interventions against this devastating genetic disorder.

## Methods

### Animals

All the mice were housed in a temperature- and humidity-controlled facility with ad libitum access to food and water. All procedures were approved by the Institutional Animal Care and Use Committee (IACUC) of the Shonan Health Innovation Park, iPark Institute Co., Ltd., accredited by AAALAC International, and conducted in accordance with institutional guidelines. All animal experiments were conducted using male mice and approved by the Institutional Animal Care and Use Committee of Shonan Research Center (AU-00031347). The study is reported in accordance with ARRIVE guidelines.

### Generation of inducible exon-skipping mouse model

Inducible exon-skipping mice were generated using the CRISPR/Cas9 system. *Col4a5* R471* floxed mice were created by introducing a targeting vector containing the R471* mutation flanked by loxP sites using polymerase chain reaction (PCR)-amplified fragments from C57BL/6 J genomic DNA. The vector, guide RNA targeting *Col4a5* (sequence: CAAGGAGAGCGAGGAGTAAAAGG), and Cas9 protein were microinjected into fertilized C57BL/6 J eggs. Similarly, Nphs2-CreERT2 mice were generated by inserting a T2A-CreERT2 cassette in-frame after leucine 385 in exon 8 of *Nphs2*. The cassette, along with the gRNA targeting *Nphs2* (sequence: TCTCCTATGTTATAGGCGAATGG) and Cas9 protein, was injected into fertilized eggs. To obtain hemizygous *Col4a5* R471* floxed male mice with an *Nphs2*-CreERT2 transgene (*Nphs2*^CreER/+^; *Col4a5*^R471^^* fl/y^), heterozygous *Col4a5* R471* floxed female mice were crossed with heterozygous *Nphs2*-CreERT2 male mice.

### Genotyping

Genotyping PCR was performed using genomic DNA extracted from the ear tissue, with specific primer sets designed for each transgenic line. For the *Col4a5* R471* floxed mouse, the primers used were 5′- TTTATCCTGACATGCTTGGCTGTTG -3′ (forward) and 5′- GAGTTGATTTCAATGAGTGGTCATGC

-3′ (reverse), generating PCR products of 681 bp for the wild-type (WT) allele and 749 bp for the *Col4a5* R471* floxed allele. Similarly, for the *Nphs2*-CreERT2 mouse, the primers used were 5′- TGTTGATAGTGTGTTCACGTGACTG -3′ (forward) and 5′- CGCGCCTGAAGATATAGAAGATAAT

 -3′ (reverse), yielding a PCR product of 1304 bp for the *Nphs2*-CreERT2 allele. Verification of the transgene sequence was conducted via direct Sanger sequencing of PCR-amplified fragments using the primers described above. Sequencing reactions were performed using a BigDye Terminator v3.1 Cycle Sequencing Kit (Thermo Fisher Scientific, Waltham, MA) and analyzed on a 3130xl Genetic Analyzer (Thermo Fisher Scientific).

### Tamoxifen administration

A tamoxifen-containing diet (250 mg/kg; Sigma-Aldrich) was formulated based on the AIN-76A diet (Research Diets, Inc., NJ, USA). The mice were divided into three groups: no tamoxifen, tamoxifen from 6 to 10 weeks of age, and tamoxifen from 14 to 18 weeks of age. Each group received tamoxifen in two one-week cycles separated by one-week intervals. WT mice were used as the controls. Six mice were used in each group. The study began at 6 weeks of age, and body weights were recorded every two weeks. Urine samples were collected at 6, 10, 14, 16, and 22 weeks of age. At 22 weeks of age, all mice were anesthetized with isoflurane (3–5%) using anesthetic vaporizer (ISOREX I-200, SHIN-EI INDUSTRIES,INC., Japan), and after it was confirmed that the animal was in a deep anesthetized state based on the pain reflex, all blood was collected from the abdominal vena cava, and the animal was euthanized by exsanguination. Then, kidneys were harvested.

The timing of tamoxifen administration was determined based on a comprehensive assessment of previous data on urinary albumin-to-creatinine ratio (UACR) and glomerulosclerosis index, which reflect the progression of renal pathology in this model, including findings at 14 weeks of age reported in a previous study^18,20^..

### Urine analysis

Urine samples were collected by housing mice individually in metabolic cages for 16 h with free access to food and water. Samples were collected every four weeks from 6 to 22 weeks of age and centrifuged at 400 × *g* for 5 min. Urinary albumin and creatinine levels were measured using an automated analyzer (LABOSPECT008, Hitachi) with an LBIS Mouse Urinary Albumin Assay Kit and L-Type Wako CRE-M (Fujifilm Wako Pure Chemicals), respectively.

### Blood chemistry

Blood was collected from the abdominal vena cava with anesthesia using isoflurane (3–5%) by heparinized syringes. Plasma was isolated through centrifugation at 7500 × *g* for 10 min. Blood urea nitrogen (BUN) and total cholesterol (TC) levels were measured using a LABOSPECT008 analyzer with L-Type Wako UN-V and L-Type Wako CHO-M (Fujifilm Wako Pure Chemicals), respectively.

### Immunohistochemistry

Fresh frozen kidneys of 3 representative animals in each group were cryosectioned at a thickness of 8 μm using a cryostat (CM3050S, Leica Biosystems, Wetzlar, Germany). The Sections were stained with FITC-conjugated anti-COL4A5 antibody (H53 and B51; CFT45325, Shigei Medical Research Institute, Japan) following the manufacturer’s protocol.

### Histopathology

Bilateral kidneys of 6 animals in each group were fixed in 10% neutral buffered formalin, embedded in paraffin, and sectioned at a thickness of 3um. The Sections were stained with hematoxylin–eosin (HE) and Periodic acid-Schiff (PAS). Collagen fibers were stained using Sirius Red (SR), followed by counterstaining with Fast Green to provide contrast. Whole-slide digital images were acquired with a slide scanner (NanozoomerS60, (Hamamatsu Photonics, Hamamatsu, Japan). The degree of glomerulosclerosis was expressed by histopathological index following the reported method using PAS staining whole slide images^21^. Briefly, 30 glomeruli that is randomly selected at regular intervals in each kidney section. These glomeruli were categorized into four grades: grade 0 for normal structure, grade 1 for < 25% tuft involvement, grade 2 for 25–50% tuft involvement, grade 3 for 50–75% tuft involvement, and grade 4 for > 75% involvement (Fig. 5E). The index was calculated as [(1 × number of glomeruli with grade 1) + (2 × number with grade 2) + (3 × number with grade 3) + (4 × number with grade 4)] × 100%/total glomeruli observed. For fibrosis area quantification, SR positive areas/total tissue area were calculated in the cortex (cortex and outer stripe of the outer medulla) or medulla (inner stripe of the outer medulla, inner medulla and papilla) using an image analysis software (HALO, Indica Labs, Corrales, NM, USA). In the analysis, renal capsule, hilar adipose tissue, and renal artery were excluded from region of interest using tissue segmentation. In addition, pelvic fibrous tissues were manually excluded by pathologist. The reason of the exclusion was that the tissue components have physiological collagen fibers and was included with various percentage in the specimens as analysis noise.

### Transmission electron microscopy (TEM)

Renal cortex samples of 2 representative animals in each group were fixed with 2.5% glutaraldehyde in 0.1 M phosphate buffer (pH 7.4) at 4 °C for 2 h., followed by post-fixation in 1 w/v% osmium tetroxide for 2 h at 4 °C. The Samples were embedded in Epon 812 resin and ultrathin sections were stained with uranyl acetate (2%, 20 min at room temperature) and lead citrate (9 min at room temperature). Sections were visualized using an H-7600 TEM (Hitachi).

### Statistical analysis

Statistical analyses were performed using EXSUS (version 10.1; EPS Corporation, Tokyo, Japan), which is integrated with SAS software (version 9.4). All data are presented as mean ± standard deviation. UACR, plasma BUN and TC levels were analyzed using Aspin-Welch’s t-test. SR staining positive area and glomerulosclerosis index were analyzed using the Steel–Dwass multiple comparison test. *p*-values < 0.05 were considered statistically significant.

## Results

### Generation of a Tamoxifen-inducible *Col4a5* R471* floxed Mouse Model Using Cre-loxP Recombination

**Fig. 1:**
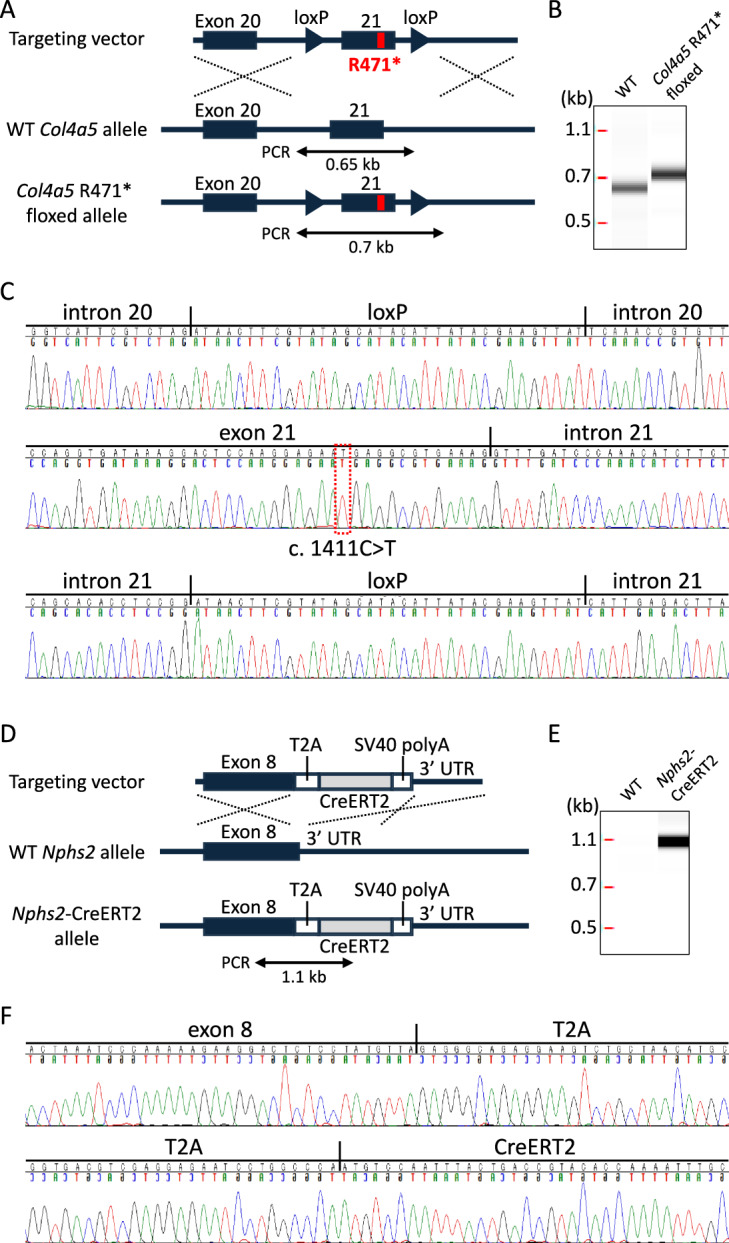
Generation of *Col4a5* R471* floxed mice and *Nphs2*-CreERT2 mice. (**A**) Schematic representation of the targeting strategy for knock-in of the *Col4a5* R471* floxed sequence at the *Col4a5* locus. (**B**) PCR analysis for *Col4a5* R471* floxed mouse. *Col4a5* R471* floxed mouse showed a 749-bp band, and wild type (WT) mouse showed a 681-bp band. (**C**) Sanger sequencing results of *Col4a5* R471* floxed allele are shown. The upper panel shows the loxP site inserted into intron 20 of the endogenous *Col4a5* gene. The middle panel displays the targeted *Col4a5* R471* mutation (c. 1411C > T) along with several silent mutations. The lower panel presents the loxP site inserted into intron 21 of the endogenous Col4a5 gene. (**D**) Schematic representation of the targeting strategy used for knock-in of a T2A-CreERT2 cassette at the *Nphs2* locus. (**E**) PCR analysis for *Nphs2*-CreERT2 mouse. *Nphs2*-CreERT2 mouse showed a 1304 bp-positive band, and wild type (WT) mouse showed no band. (**F**) Sanger sequencing results of *Nphs2*-CreERT2 allele are shown. The sequence shown includes the last 34 bases of exon 8 of the endogenous *Nphs2* gene, the complete T2A sequence, and the first 34 bases of CreERT2.

To develop a conditional model of X-linked AS, we engineered a *Col4a5* R471* floxed allele that enabled exon 21 to skip upon Cre-mediated recombination. As illustrated in Fig. 1A, the targeting vector was designed to introduce loxP sites flanking exon 21, which harbors the R471* nonsense mutation. The genomic structures of the WT *Col4a5* allele (Fig. 1B) and the successfully modified *Col4a5* R471* floxed allele (Fig. 1C) were confirmed using PCR-based genotyping and sequencing, which verified the correct insertion of both loxP sites without disrupting the flanking intronic regions. To achieve podocyte-specific and temporally controlled recombination, we generated *Nphs2*-CreERT2 mice, in which the CreERT2 cassette was inserted downstream of the endogenous *Nphs2* gene via a T2A self-cleaving peptide (Fig. 1D). This design enables the bicistronic expression of endogenous podocin and CreERT2 while preserving normal Nphs2 expression. The WT *Nphs2* allele (Fig. 1E) and CreERT2 knock-in allele (Fig. 1F) were validated using PCR with allele-specific primers, confirming appropriate recombination at the intended locus.

### Establishment of a tamoxifen-inducible *Col4a5* Exon 21 skipping mouse model

**Fig. 2:**
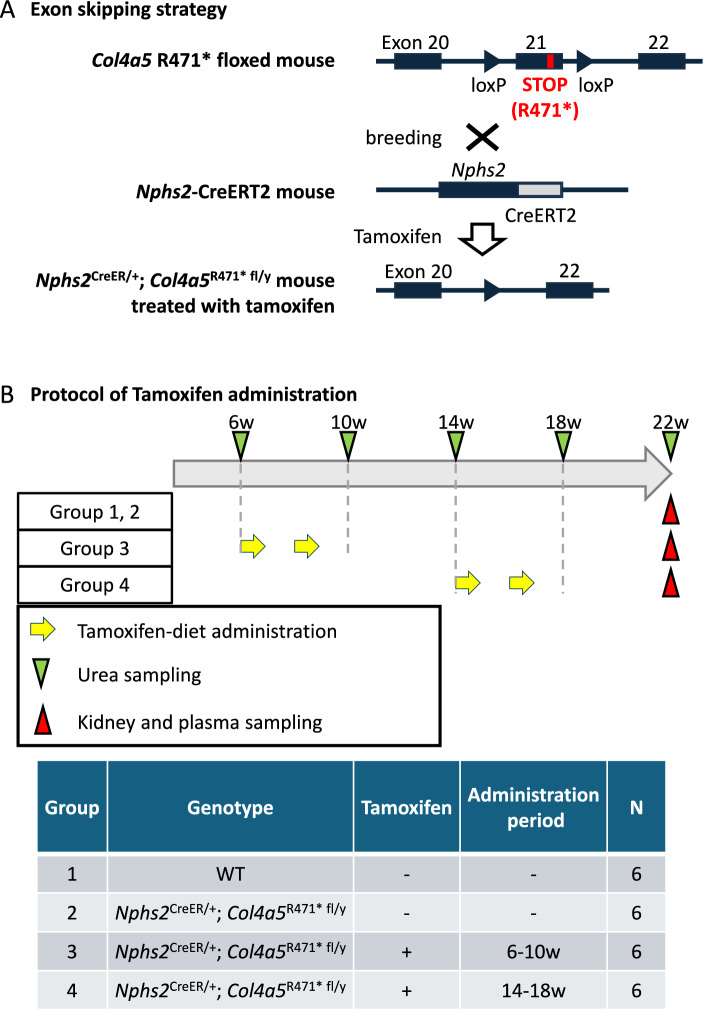
Generation and experimental design of a tamoxifen-inducible *Col4a5* exon 21-skipping Alport syndrome mouse model. (**A**) Schematic representation of the genetic engineering strategy to create conditional exon 21-skipping mice. *Col4a5* R471* floxed mice carry a nonsense mutation (c.1411C > T, p.R471*) in exon 21 and two loxP sites flanking exon 21 of the *Col4a5* gene. These mice were crossed with *Nphs2*-CreERT2 mice, which express a tamoxifen-inducible Cre recombinase (CreERT2) under the control of the podocyte-specific *Nphs2* promoter. Tamoxifen administration induces Cre-mediated deletion of exon 21 in glomerular podocytes, resulting in in-frame exon skipping and expression of a truncated but partially functional COL4A5 protein. (**B**) Experimental protocol for tamoxifen administration and sample collection. Mice were grouped according to genotype and timing of tamoxifen exposure. Group 1: wild type (WT, no tamoxifen), Group 2: *Nphs2*^CreER/+^; *Col4a5*^R471^^* fl/y^ (no tamoxifen), Group 3: tamoxifen administered from 6 to 10 weeks (early intervention), and Group 4: tamoxifen administered from 14 to 18 weeks (therapeutic intervention). Tamoxifen-diet was administrated in two cycles of one week on and one week off for four weeks. All mice were sacrificed at 22 weeks of age for kidney and plasma sampling. Tamoxifen was delivered via a 0.025% tamoxifen-containing diet. Urea and urine samples were collected at defined intervals for functional analysis.

To investigate the therapeutic potential of exon skipping after disease onset in AS, we developed a conditional exon 21-skipping mouse model. We generated AS model mice harboring a point mutation in exon 21 of *Col4a5* (c.1411C > T, resulting in a premature stop codon at amino acid position 471, R471*) and inserted two loxP sites flanking exon 21 (hereafter referred to as *Col4a5* R471* floxed mice). These loxP sites enabled the Cre recombinase–mediated excision of exon 21, allowing temporal control of exon skipping (Fig. 2A, upper panel). Upon administration of tamoxifen, CreERT2 is activated in glomerular podocytes and mediates the excision of exon 21 in the *Col4a5* transcript. This leads to the production of an internally truncated COL4A5 protein lacking exon 21, effectively mimicking the mechanism of exon-skipping ASO therapy. Importantly, this system enabled precise temporal control of exon skipping, allowing us to investigate both prophylactic and therapeutic interventions depending on the timing of tamoxifen administration (Fig. 2A, lower panel). The experimental protocol for tamoxifen administration is illustrated in Fig. 2B.

### Physiological and biochemical improvements following induced exon 21 skipping in *Col4a5*

To evaluate the therapeutic efficacy of *Col4a5* exon 21 skipping on disease progression in AS model mice, we monitored multiple physiological and biochemical parameters across all experimental groups. Body weight, UACR, and plasma levels of BUN and TC were measured longitudinally or at the endpoint to assess the overall health status and kidney function.

**Fig. 3:**
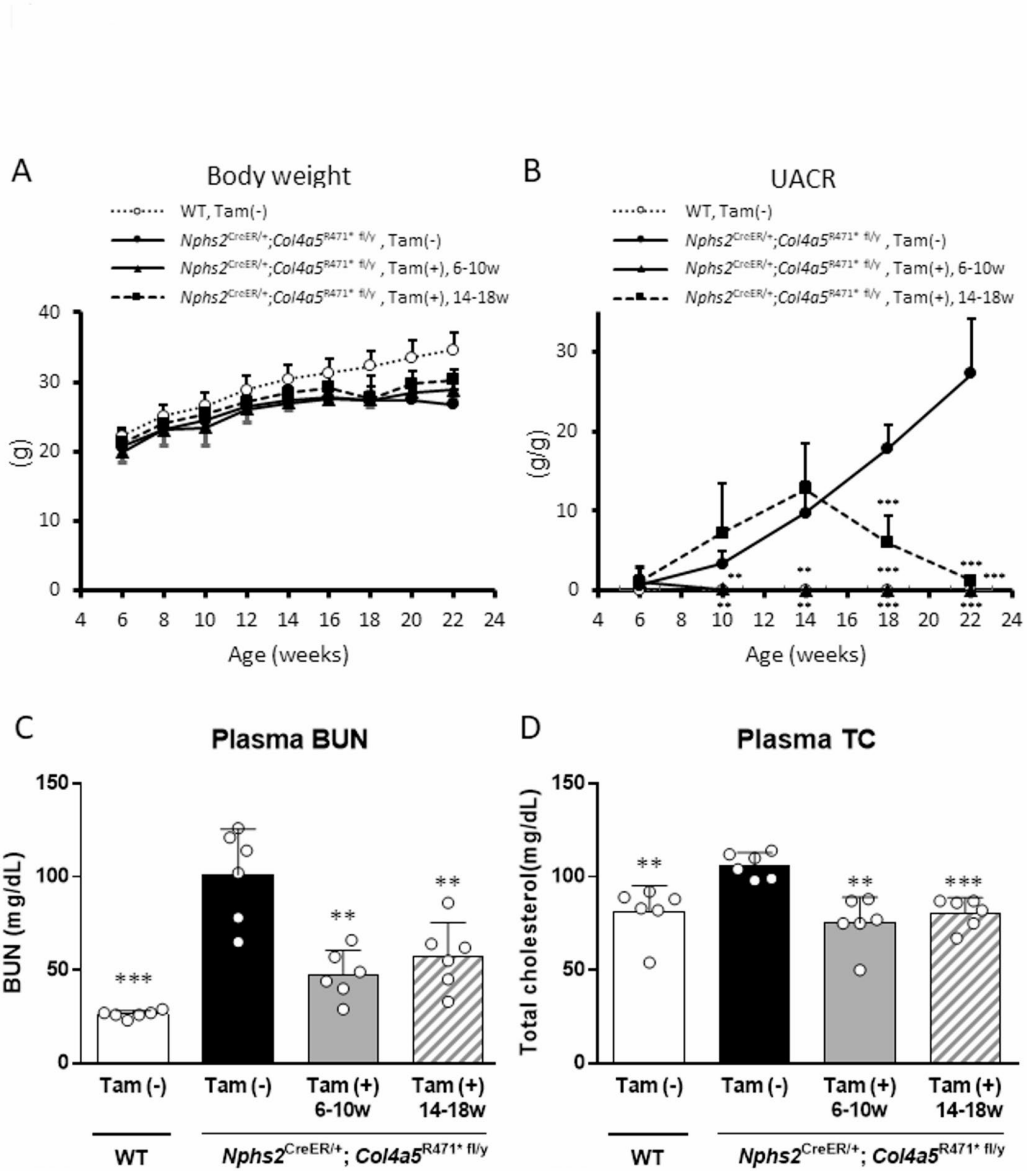
Effects of tamoxifen-induced *Col4a5* exon 21 skipping on body weight, urinary albumin-to-creatinine ratio, and plasma biomarkers in Alport syndrome model mice. (**A**) Longitudinal body weight measurements from 6 to 22 weeks of age. Untreated AS model mice (*Nphs2*^CreER/+^; *Col4a5*^R471* fl/y^, Tam ( −)) showed mildly reduced growth compared with wild-type controls. Both early (6–10 weeks) and late (14–18 weeks) tamoxifen-treated groups exhibited partial restoration of body weight. (**B**) Urinary albumin-to-creatinine ratio (UACR) as a marker of glomerular injury. Untreated AS model mice showed significantly elevated UACR beginning at 8 weeks of age. Tamoxifen-induced exon skipping from both early and late time points significantly reduced UACR to levels comparable to wild-type mice. (**C**) Plasma blood urea nitrogen (BUN) levels measured at 22 weeks. Elevated BUN in untreated AS mice was significantly decreased following tamoxifen administration in both early and late treatment groups. (**D**) Plasma total cholesterol (TC) levels measured at 22 weeks. Hypercholesterolemia in AS model mice was ameliorated by tamoxifen-induced Col4a5 exon skipping, with significant reductions in both treatment groups. **: p < 0.01, ***: p < 0.001 in *Nphs2*^CreER/+^; *Col4a5*^R471* fl/y^ mice treated with normal chow vs. other groups by Aspin-Welch t-test. Alb: albumin, CRE: creatinine, BUN: blood urea nitrogen. Data were shown as mean + SD.

Body weight was measured biweekly from 6 to 22 weeks of age in all four experimental groups: WT, AS model mice without tamoxifen treatment (*Nphs2*^CreER/+^; *Col4a5*^R471* fl/y^, Tam ( −)), prophylactically treated mice (tamoxifen administered from 6 to 10 weeks), and therapeutically treated mice (tamoxifen administered from 14 to 18 weeks). All the groups showed steady growth with age. However, AS model mice without tamoxifen treatment exhibited a mild but consistent reduction in body weight compared with WT controls, suggesting chronic kidney disease-associated growth retardation. In contrast, both the prophylactic and therapeutic tamoxifen-treated groups showed partial recovery of the body weight trajectory toward normal levels, implying an improved overall health status following *Col4a5* exon skipping (Fig. 3A).

UACR is a sensitive indicator of glomerular injury. In untreated AS model mice, the UACR began to increase significantly by 8 weeks of age and continued to increase over time, indicating progressive glomerular dysfunction. Tamoxifen-induced skipping of *Col4a5* exon 21 from 6 weeks of age markedly suppressed this increase, maintaining UACR levels close to those observed in WT mice. The therapeutic administration of tamoxifen from 14 weeks of age, after the onset of proteinuria, also resulted in a substantial reduction in UACR by 22 weeks, effectively reversing the progression of proteinuria in most animals. These findings suggest that both early and late exon skipping in mutant *Col4a5* significantly protected against glomerular barrier breakdown and protein loss in the urine (Fig. 3B).

Plasma BUN levels, a marker of nitrogenous waste accumulation due to impaired renal excretory function, were significantly elevated in the untreated AS model mice at 22 weeks. In both tamoxifen-treated groups, BUN levels were significantly reduced, approaching those of the WT mice. This effect was observed regardless of whether tamoxifen was administered before or after disease onset, supporting the idea that restoring partially functional COL4A5 protein levels can improve renal clearance and reduce uremic toxin build-up (Fig. 3C).

Hypercholesterolemia is often associated with proteinuric nephropathies. Consistent with this finding, the untreated AS model mice displayed significantly increased plasma TC levels at 22 weeks. Both prophylactic and therapeutic tamoxifen treatments led to significant reductions in plasma cholesterol levels, further supporting the therapeutic benefit of *Col4a5* exon 21 skipping in ameliorating the systemic consequences of proteinuria and CKD (Fig. 3D).

### Restoration of type IV collagen α5 chain expression in the kidney following tamoxifen-induced exon skipping

To confirm whether tamoxifen-induced exon 21 skipping in *Col4a5* leads to the production and localization of a truncated but functional COL4A5 protein, we performed immunofluorescence staining of kidney sections collected at 22 weeks of age. This analysis allowed visualization of the α5 chain of type IV collagen, the key component of the GBM and tubular basement membrane, whose absence underlies the structural dysfunction in AS.

**Fig. 4:**
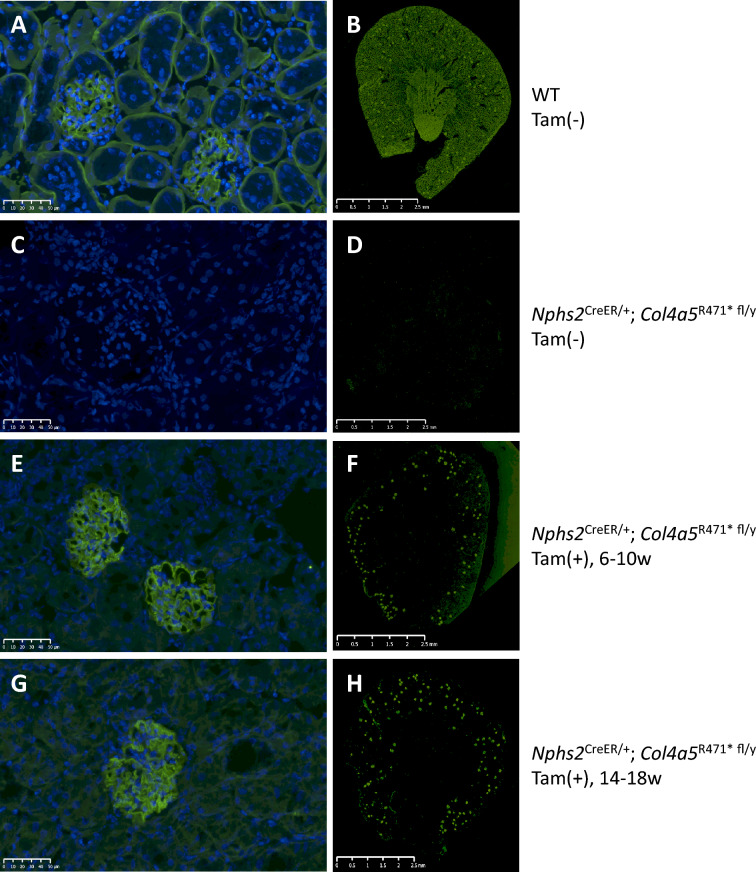
Restoration of type IV collagen α5 chain expression in the kidney of Alport syndrome model mice following tamoxifen-induced exon 21 skipping. Representative histological images from n = 3 mice per group. Immunofluorescence staining for the α5 chain of type IV collagen (COL4A5) in kidney sections collected at 22 weeks of age. (**A**, **B**) Wild-type mice (WT, Tam ( −)) showed strong and continuous COL4A5 expression in both the glomerular and tubular basement membranes. (**C**, **D**) AS model mice without tamoxifen treatment (*Nphs2*^CreER/+^; *Col4a5*^R471* fl/y^, Tam ( −)) exhibited complete loss of COL4A5 expression in all renal compartments, confirming the absence of functional COL4A5 protein. (**E**, **F**) In mice receiving tamoxifen from 6 to 10 weeks of age (*Nphs2*^CreER/+^; *Col4a5*^R471* fl/y^, Tam ( +), 6–10w), corresponding to the prophylactic treatment group, global and diffuse recovery of COL4A5 expression was observed in the glomerular basement membrane. (**G**, **H**) In mice treated with tamoxifen from 14 to 18 weeks of age (*Nphs2*^CreER/+^; *Col4a5*^R471* fl/y^, Tam ( +), 14–18w), corresponding to the therapeutic treatment group, COL4A5 protein expression was restored in the glomeruli at 22 weeks despite ongoing disease progression. The fluorescence signals were primarily localized to the glomerular basement membrane, which is consistent with podocyte-specific recombination. Images are representative of 6 mice per group. Sections were stained with an FITC-conjugated anti-COL4A5 antibody (H53 and B51; CFT45325; Shigei Medical Research Institute, Okayama, Japan). Bar = 1 µm, CL: glomerular capillary lumen.

In WT mice, robust and continuous expression of the COL4A5 protein was observed along the GBM and tubular basement membrane, consistent with normal renal architecture (Fig. 4A and B). The staining pattern was linear and intense in both glomerular and tubular regions, indicating the presence of a fully assembled α3α4α5 collagen network. In contrast, *Nphs2*-CreERT2/*Col4a5* R471* floxed mice without tamoxifen treatment showed a complete absence of COL4A5 staining in all renal compartments, including both glomeruli and tubules (Fig. 4C and D). This confirmed the successful disruption of COL4A5 expression in our AS model and recapitulated the hallmark molecular pathology of XLAS.

Remarkably, mice treated with tamoxifen from 6 to 10 weeks of age (prophylactic group) exhibited partial restoration of COL4A5 expression in the glomeruli (Fig. 4E and F). Staining was detectable and localized along the GBM, although the intensity and distribution were slightly reduced compared with those in WT mice, likely reflecting the truncated nature of the protein resulting from exon 21 skipping. Importantly, no ectopic or diffuse staining was observed, suggesting that the protein was correctly targeted to GBM. Similarly, in mice treated with tamoxifen from 14 to 18 weeks of age (therapeutic group), COL4A5 expression was restored in the glomeruli by the endpoint at 22 weeks (Fig. 4G and H). The reappearance of α5(IV) collagen in the GBM in this group was particularly significant, as it occurred despite the presence of ongoing renal injury, indicating that the genetic rescue of COL4A5 expression is feasible even after disease onset.

In both treatment groups, COL4A5 recovery appeared to be limited primarily to the glomeruli, with minimal or no detectable restoration of the tubular basement membrane. This is consistent with the podocyte-specific activity of the Nphs2 promoter driving CreERT2 and highlights the importance of podocyte-expressed collagen in GBM integrity.

### Histological changes in kidney and fibrosis analysis by sirius red staining

**Fig. 5:**
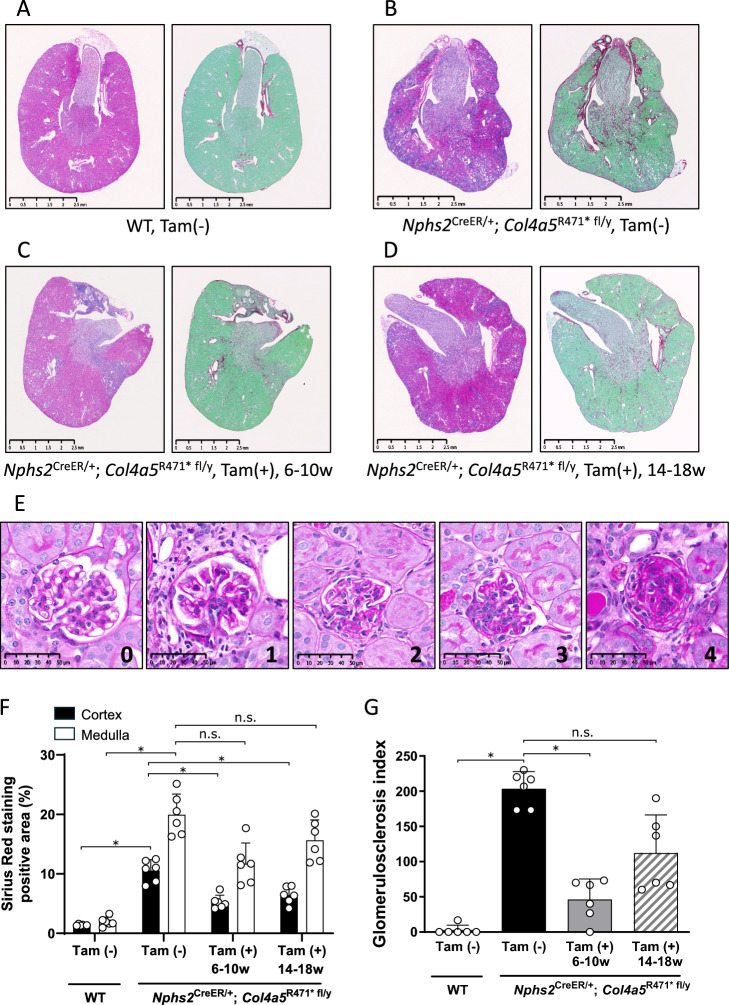
Collagen 4a5 recovery ameliorates renal pathology in AS model mice. (**A**–**D**) Representative histological images from n = 6 mice per group. HE-stained panels are shown on the left, and SR-stained panels are shown on the right. Green counterstain in SR images highlights tissue structure and is described in detail in the Methods section. (**A**) Wild-type kidneys show normal histology with a smooth surface, absence of basophilic areas, and no SR positive regions, indicating healthy renal morphology. (**B**) In contrast, kidneys from *Nphs2*^CreER/+^; *Col4a5*^R471* fl/y^ mice, which lack COL4A5, exhibit significant pathological changes, including regenerative, sclerotic, and fibrotic lesions in both the glomeruli and tubulointerstitial areas. These lesions are characterized by a rough surface, basophilic appearance, and increased SR positive areas. (**C**, **D**) Following tamoxifen treatment in both prophylactic and disease-onset phases, COL4A5 protein recovery leads to a marked reduction in lesion size and a localized distribution of collagen. This indicates an amelioration of the AS phenotype, with significant improvements in kidney pathology, including a reduction in fibrosis and sclerosis. These findings suggest that COL4A5 recovery contributes to the restoration of kidney function and structure in the AS model mice. (**E**) High magnification of the glomeruli showing grade 0 to 4 for glomerulosclerosis index. The images of glomeruli are captured from Wild-type mice (grade 0), AS model mice treated with tamoxifen from 14 to 18 weeks of age (grade 1–3), and AS model mice without tamoxifen treatment (grade 4). (**F**) Quantification of fibrotic area by SR staining and (**G**) glomerulosclerosis index at 22 weeks of age. Statistical analysis was performed using the Steel–Dwass multiple comparison test. *: p < 0.05, *Nphs2*^CreER/+^; *Col4a5*^R471* fl/y^ mice treated with normal chow vs. other groups.

To evaluate the ameliorative effects of COL4A5 on protein recovery in kidneys, we performed semi-quantitative or quantitative histopathological analysis. Generally, no significant abnormalities were observed in the WT kidney tissue (Fig. 5A). In contrast, kidneys from *Nphs2*-CreERT2/*Col4a5* R471* floxed mice, which lack COL4A5 protein, exhibited clear signs of pathological changes, including regenerative, sclerotic, and fibrotic lesions in both the glomeruli and tubulointerstitial areas (Fig. 5B). These lesions were marked by an irregular rough surface, basophilic staining, and increased SR-positive areas, indicating severe renal damage associated with the absence of COL4A5. Following tamoxifen treatment, we observed a significant recovery in COL4A5 protein expression in both the prophylactic and disease-onset treatment phases. The treatment resulted in the reduction of fibrotic and sclerotic lesions, with the affected areas becoming smaller and demonstrating a more localized distribution of collagen (Fig. 5C and D).

In addition, the results of the glomerulosclerosis index and fibrosis image analysis suggested that amelioration by treatment with tamoxifen especially in the renal cortex (Fig. 5E-G). Fibrosis image analysis revealed that the percentage of fibrosis area was increased both in the cortex and medulla in mice without tamoxifen compared to WT mice. Interestingly, the fibrosis area of the cortex was significantly decreased with tamoxifen treatment. On the other hands, the fibrosis area of the medulla was not significantly decreased with tamoxifen treatment that was suggested amelioration of disease condition more in the cortex than medulla. The result was correlated with the restoration of COL4A5 expression only in the glomeruli. The glomerular sclerosis index was significantly increased in AS model mice without tamoxifen compared to WT mouse. With tamoxifen treatment, the index was decreased and statistically decreased in mice treated with tamoxifen from 6 to 10 weeks of age. These results suggest that the recovery of COL4A5 contributes to amelioration of the AS phenotype, improving both the structural integrity and function of the kidneys.

### GBM recovery analysis using TEM

**Fig. 6:**
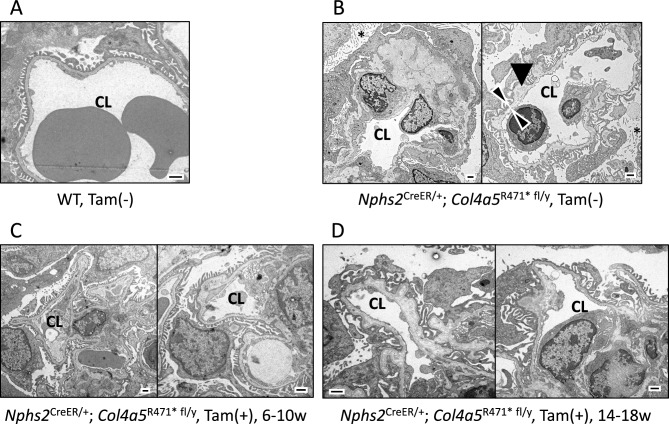
Transmission electron microscopy (TEM). (**A**- **D**) Representative TEM images from n = 2 mice per group. (**A**) Wild-type mice display a normal glomerular basement membrane (GBM) structure with no signs of pathology, indicating healthy renal morphology. (**B**) In contrast, *Nphs2*^CreER/+^; *Col4a5*^R471* fl/y^ mice, which lack COL4A5, show significant GBM abnormalities, including irregular thickening or thinning, splitting (arrowheads), electron-dense granules (arrow), and lucent areas in the GBM. These structural defects are accompanied by foot process effacement and increased microvilli (asterisk) on the podocytes, indicating disruption of the filtration function. (**C**, **D**) After tamoxifen treatment, both during the prophylactic and disease-onset phases, recovery of collagen 4a5 expression leads to amelioration of these pathological features. The GBM structure is partially restored, and foot process effacement is reduced, demonstrating that COL4A5 recovery improves podocyte integrity and preserves kidney filtration function in the AS model mice. These lesions are accompanied by foot process effacement and increased microvilli (asterisk) of the podocytes. Moreover, the lesions are localized and mildly observed in the Tam ( +), 6–10 w or 14–18 w group. Bar = 1 µm, CL: glomerular capillary lumen.

To further investigate the ameliorative effects of COL4A5 protein recovery in the kidneys of AS model mice, we used TEM to examine the GBM structure. In WT mice, the GBM appeared intact, showing no signs of pathological alterations (Fig. 6A). In contrast, in *Nphs2*-CreERT2/*Col4a5* R471* floxed mice, which lack COL4A5, we observed significant structural abnormalities in the GBM (Fig. 6B). These included irregular thickening or thinning of the membrane, splitting (indicated by arrowheads), electron-dense granules (denoted by arrows), and lucent areas within the GBM. These changes were accompanied by substantial foot process effacement and an increase in microvilli (indicated by asterisks) in the podocytes, which are crucial cells involved in kidney filtration. These ultrastructural abnormalities highlight the detrimental impact of COL4A5 deficiency, leading to podocyte loss and subsequent disruption of kidney filtration function. However, after tamoxifen treatment, both in the prophylactic (Fig. 6C) and disease-onset phases (Fig. 6D), we observed a significant recovery of COL4A5 expression, which in turn led to a noticeable amelioration of these GBM and podocyte abnormalities.

## Discussion

AS is a hereditary kidney disorder that is characterized by progressive glomerulopathy, sensorineural hearing loss, and ocular abnormalities. The development of various animal models of AS, particularly mice and dogs, has significantly contributed to the understanding of disease mechanisms and preclinical evaluation of potential therapies^18,22–28^. Two early *Col4a3* knockout models were described in 1996. One was created by inserting a neomycin cassette into exon 48 of *Col4a3*^25^, while the other involved the deletion of three exons between exons 48 and 50^26^. Both models resembled autosomal recessive AS in humans. Additionally, a double knockout of *Col4a3* and *Col4a4* was generated by a large deletion between exon 2 of *Col4a3* and exon 12 of *Col4a4*^27^. This double knockout mice was generated and maintained on the FVB/N genetic background, which likely explains the observed severity. The FVB/N strain is known to be highly susceptible to glomerular disease, as demonstrated in Cd151-deficient mice^29^. A further model involving a *Col4a5* knockout was generated by a nonsense mutation in exon 1 of *Col4a5*^28,30^. While most existing AS mouse models involve complete knockouts of collagen IV α-chains, such as *Col4a3* or *Col4a5*, these do not perfectly replicate the genetic heterogeneity observed in human patients.

Human AS is primarily caused by various mutations, including point mutations, insertions, deletions, or splicing variants in the *COL4A3*, *COL4A4*, or *COL4A5*genes, with no clear mutational hotspot^31^. This variability underscores the need for mouse models that harbor patient-specific mutations to evaluate mutation-targeted therapies, such as exon skipping or mRNA replacement approaches. Furthermore, many existing knockout models exhibit rapid disease progression and short lifespans, limiting their utility for evaluating long-term therapeutic interventions. For instance, *Col4a3* knockout mice on the 129/Sv background typically develop end-stage renal disease more rapidly than *Col4a5* G5X mice on the C57BL/6.Cg background. *Col4a3*knockout mice on the 129/Sv background died at approximately 14 weeks of age^25,26^, while *Col4a5*G5X mice on the C57BL/6.Cg background died by approximately 22 weeks^26,28^. Considering the phenotypes of AS model mice, we should realize there are differences of genetic mutation and strain-related differences. In response to these limitations, we previously developed a novel AS model with a patient-derived nonsense mutation (c.1411C > T, p.R471*) in exon 21 of *Col4a5*, which mimics a known human XLAS mutation^33^. This model, with a pure C57BL/6 (B6) background, provides more consistent phenotypic comparisons with other B6-based disease models, avoiding the confounding effects of mixed genetic backgrounds, such as the commonly used 129/Sv strains. Our R471* model demonstrated a significantly extended lifespan of up to 28 weeks, enabling a detailed longitudinal analysis of disease progression and therapeutic responses, which is crucial for translational research.

The phenotypic features of this model mirror those of human AS, including progressive proteinuria, glomerulosclerosis, and tubulointerstitial fibrosis. TEM revealed characteristic GBM abnormalities such as irregular thickening, lamellation, and splitting, similar to human pathology. R471* mice progress to end-stage renal disease more slowly than existing models, providing a more appropriate time window for therapeutic interventions that better mimic the human clinical course^18^. However, unlike human AS, in which hematuria typically presents at an early stage, hematuria in the R471* model was not observed until 22 weeks of age^18^. This delayed onset represents a limitation in the translational applicability of the model, particularly for studying early disease markers. Similar patterns of renal fibrosis and GBM pathology are also observed in 10–30% of human CKD cases not associated with metabolic comorbidities such as diabetes or hypertension^34^. Thus, this model is not only a valuable tool for studying AS but could also aid in investigating CKD mechanisms unrelated to metabolic etiologies.

There are currently no curative treatments for AS. Standard care includes angiotensin-converting enzyme inhibitors or ARBs, which can delay disease progression but do not address the underlying genetic defects. Recently, anti–miR-21 therapy administered by subcutaneous injection in *Col4a3*knockout mice led to an improved kidney phenotype, with reductions in BUN, albuminuria, glomerulosclerosis, interstitial fibrosis, tubular injury, and inflammation, correlating with prolonged survival and preserved renal function^35^. Gene expression of miR-21 is upregulated in kidney and anti-miR-21 enhances PPARα activities which stimulates healthy mitochondrial functions through suppression of ROS production in podocyte and inhibits NF-κB and TGF-β signaling for anti-fibrotic and anti-inflammatory effects^35^. miR-21 expression is confirmed in broad tissue beyond kidney including liver, heart, injured tissue and cancer tissue. Therefore, systemic suppression of miR-21 should be focused on side effects on various tissue. Long term evaluation for safety and confirmation on generation of neutralizing antibody for anti-miR-21 might be needed. Gene therapy, including viral vector-based approaches or recombinant protein replacement, holds promise but faces significant challenges such as immunogenicity, delivery efficiency, and long-term safety^36^..

Nonetheless, some progress has been made in genetic therapies for AS. Heidet et al. have demonstrated that a human-mouse chimera of the alpha3-alpha4-alpha5(IV) collagen protomer rescued the renal phenotype in *Col4a3*^-/-^AS mice^37^. Additionally, an inducible chimeric human/mouse *COL4A3* transgene prolonged the lifespan of *Col4a3*^-/-^mice by expressing α3, α4, and α5 (IV) chains in the GBM^38^. Heikkilä et al. have shown that renal artery perfusion with an adenoviral vector carrying human *COL4A5*cDNA in pigs resulted in successful expression of COL4A5 in the glomeruli and efficient deposition in the GBM^39,40^. Given the genetic heterogeneity of human AS, exon skipping has emerged as a promising strategy, exemplified by Food and Drug-approved therapies for Duchenne muscular dystrophy^41^. Nozu et al. have demonstrated that exon skipping ASOs targeting *Col4a5* exon 21 could be delivered to podocytes and tubular epithelial cells, improving AS phenotypes in *Col4a5*R471* mutation mice^18,19^..

In our study, we generated a podocyte-specific tamoxifen-inducible exon 21 skipping model by crossing R471* floxed mice with *Nphs2*-CreERT2 mice. This enabled the selective induction of exon skipping in *Col4a5* within podocytes at defined time points, mimicking early (slightly elevated UACR at six weeks) and late disease stages (significantly elevated UACR at 14 weeks). Although our model differs from the conventional *Col4a5*R471* mouse model used in previous studies, which lacks conditional control, the timing of intervention was informed by pathological findings reported in that model, where significant glomerular injury was observed at 14 weeks of age^20^..

Tamoxifen administration prior to or after disease onset successfully induced exon skipping, restored truncated COL4A5 expression, and improved renal outcomes. In the late intervention group, albuminuria was reduced to levels comparable to those in WT mice, and serum BUN and cholesterol levels significantly improved. Histopathological analysis and electron microscopy confirmed the amelioration of glomerular and tubular injury, indicating that the treatment not only halted further damage but also partially reversed the pathological changes. These findings suggest that the presence of residual functional glomeruli may contribute to the observed therapeutic effects, even when exon skipping is initiated at a late stage. The reduction in albuminuria following late intervention implies that remaining nephrons retain sufficient responsiveness to restore filtration barrier integrity, thereby slowing disease progression. This highlights the importance of identifying and preserving functional glomeruli as a key factor in optimizing the timing and efficacy of therapeutic interventions in AS. These insights align with our observation that glomerulosclerosis could be suppressed when COL4A5 expression was restored before a critical threshold, reinforcing the concept of a therapeutic window for intervention.

Importantly, our findings emphasize that exon skipping has therapeutic potential in progressive stages of disease. This aligns with clinical expectations, where patients are often diagnosed after the onset of proteinuria or structural injury. Late interventions could therefore still provide substantial benefit, a key consideration for translating exon skipping therapies into clinical practice.

There are, however, several limitations to our study. First, the findings were obtained exclusively in a mouse model with a single patient-derived mutation and may not reflect the full genetic and phenotypic heterogeneity of human AS. Recently, Billy G. Hudson investigated that there were differences between mouse and other species such as shark and frog on the distributions of collagen 4α3, α4 and α5 chains in kidney of GBM^42^. Therefore, the therapeutic effect of exon skipping observed in mice in this study may differ in human depending on the distribution of collagen 4a5 in GBM. It would be useful to confirm the difference in distribution between mice and humans in GBM before conducting clinical trials using exon skipping. In AS, the glomerular basement membrane (GBM) of the kidney and strial capillary basement membrane (SCBM) in the inner ear, lacks the physiological association of three type IV collagen chains (α3, α4, and α5). Dominic C et al. showed collagen 4a3 deficient mice showed hearing loss^43^. Therefore, the collagen 4a5 mutation used in this experiment may also result in hearing loss, similar to that in humans but it needs confirmation the species differences in the future study. Also, it is thought that exon skipping therapy may lead to the treatment of hearing loss. Second, exon skipping in our model was restricted to podocytes, whereas other renal cell types may also contribute to disease progression and therapeutic response. Third, the truncated COL4A5 protein restored by exon skipping may only be partially functional, and its long-term safety remains to be established. Fourth, extra-renal manifestations, including hearing loss and ocular abnormalities, were not evaluated. Finally, the duration of observation was relatively short, and longer-term studies are needed to determine whether exon skipping can sustainably delay end-stage kidney disease.

In summary, our results demonstrate that exon skipping therapy can ameliorate renal pathology and restore function even after disease onset. This supports the feasibility of antisense oligonucleotide–based therapies for AS and underscores the need for further studies to optimize delivery, evaluate safety, and expand applications to other monogenic kidney diseases.

## Supplementary Information

Supplementary Information.

## Data Availability

The data supporting the findings of this study are included in this published article. Raw data generated and/or analyzed during the current study are available from the corresponding author, upon reasonable request.
