# Supplementary material for: Podocyte specific exon skipping after disease onset improves kidney pathology and function in a mouse model of Alport syndrome
Source: Sci Rep. 2025 Nov 25;15:41766. doi: 10.1038/s41598-025-25447-w (PMC12647216; doi:10.1038/s41598-025-25447-w)
Supplement: Supplementary file 1 — Supplementary Information. [file 41598_2025_25447_MOESM1_ESM.pptx]

## Slide 1
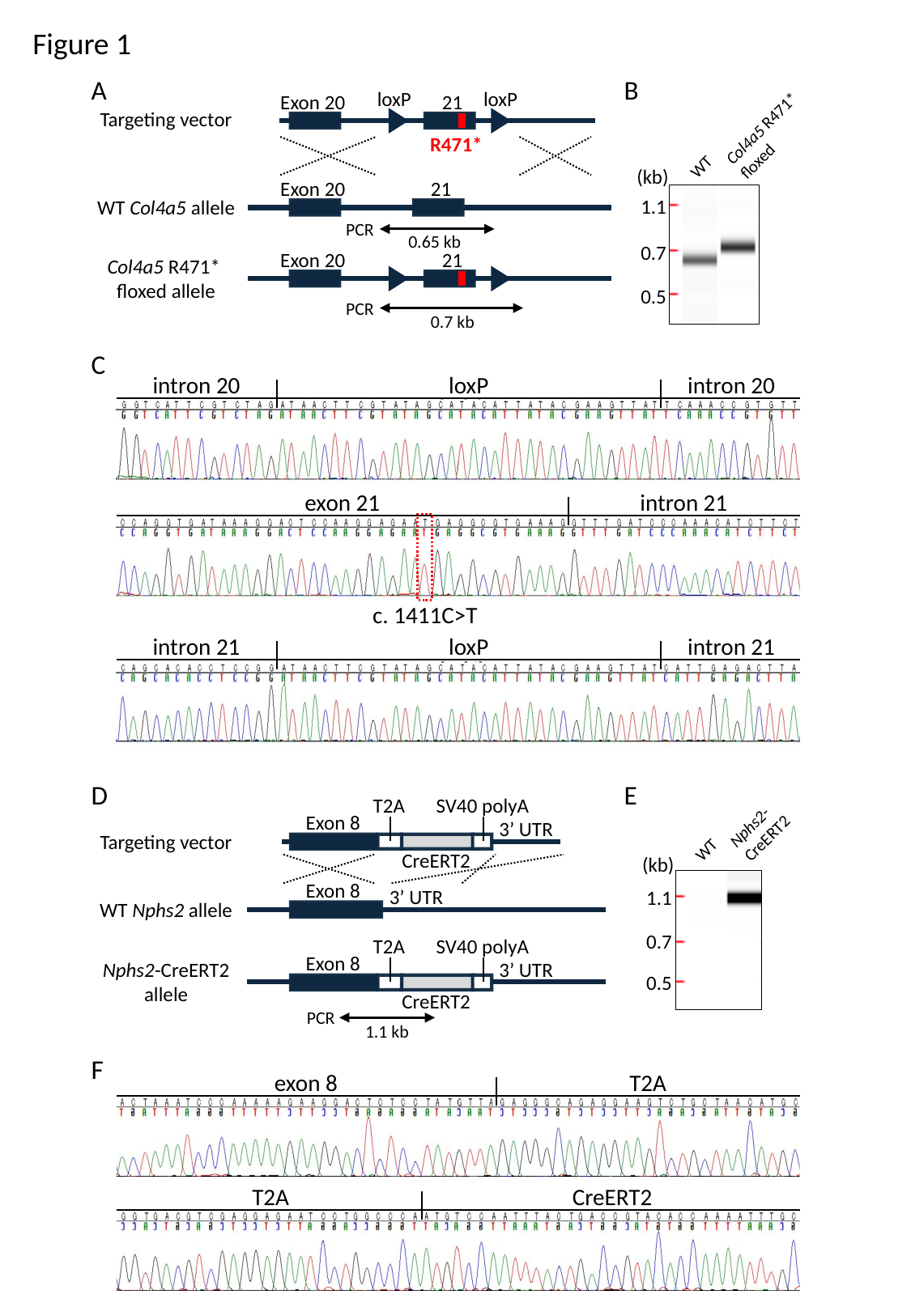

Figure 1
A
B
loxP
loxP
Exon 20
21
Targeting vector
Col4a5 R471*
floxed
R471*
WT
(kb)
1.1
0.7
0.5
Exon 20
21
WT Col4a5 allele
PCR
0.65 kb
Exon 20
21
Col4a5 R471*
floxed allele
PCR
0.7 kb
C
intron 20
loxP
intron 20
exon 21
intron 21
c. 1411C>T
intron 21
loxP
intron 21
D
E
T2A
SV40 polyA
Exon 8
Nphs2-
CreERT2
3’ UTR
Targeting vector
WT
CreERT2
(kb)
1.1
0.7
0.5
Exon 8
3’ UTR
WT Nphs2 allele
T2A
SV40 polyA
Exon 8
Nphs2-CreERT2
allele
3’ UTR
CreERT2
PCR
1.1 kb
F
exon 8
T2A
T2A
CreERT2

## Slide 2
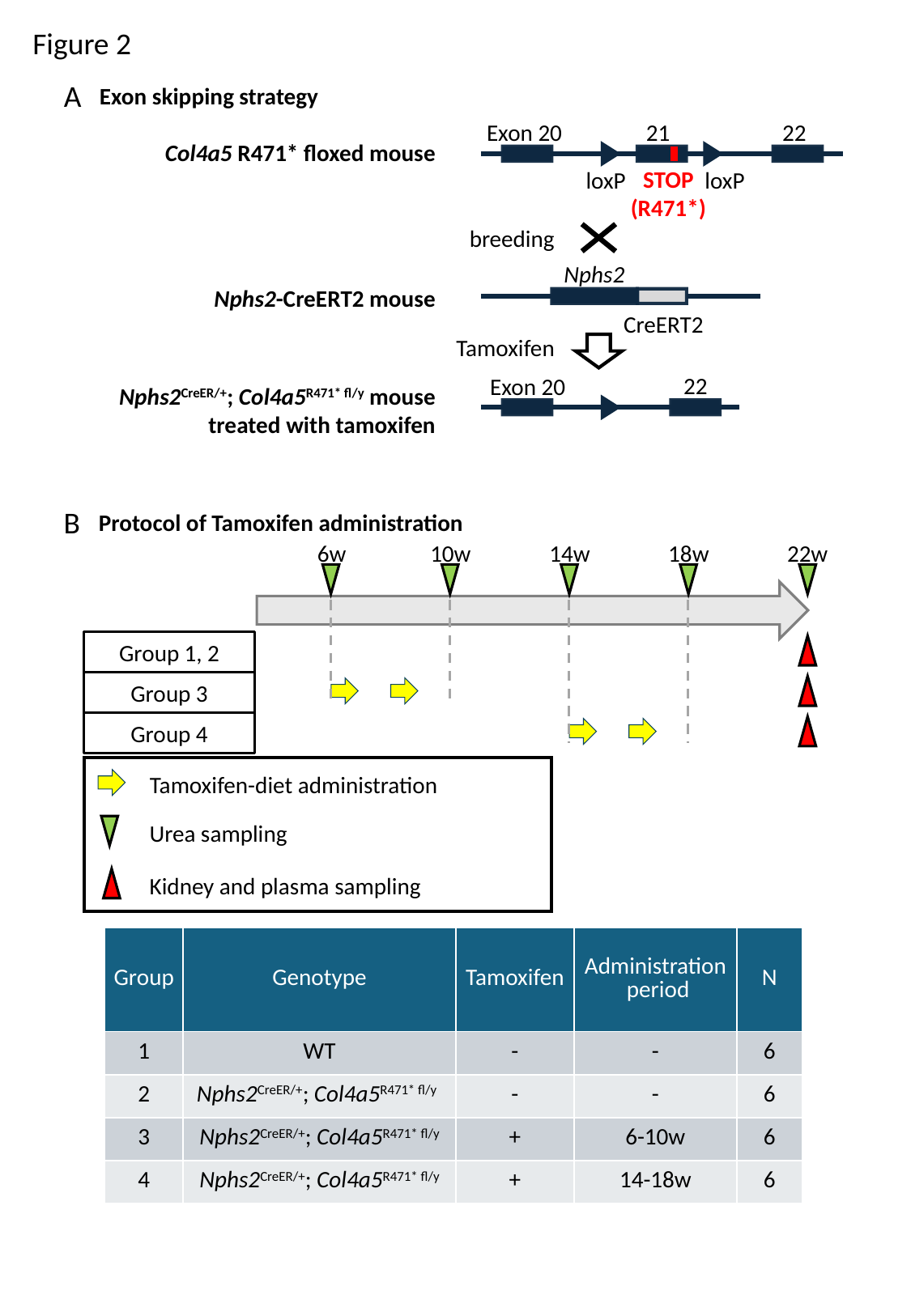

Figure 2
A
 Exon skipping strategy
Exon 20
21
22
Col4a5 R471* floxed mouse
STOP
(R471*)
loxP
loxP
breeding
Nphs2
Nphs2-CreERT2 mouse
CreERT2
Tamoxifen
22
Exon 20
Nphs2CreER/+; Col4a5R471* fl/y mouse
treated with tamoxifen
B
 Protocol of Tamoxifen administration
6w
10w
14w
18w
22w
Group 1, 2
Group 3
Group 4
Tamoxifen-diet administration
Urea sampling
Kidney and plasma sampling
| Group | Genotype | Tamoxifen | Administration period | N |
| --- | --- | --- | --- | --- |
| 1 | WT | - | - | 6 |
| 2 | Nphs2CreER/+; Col4a5R471\* fl/y | - | - | 6 |
| 3 | Nphs2CreER/+; Col4a5R471\* fl/y | + | 6-10w | 6 |
| 4 | Nphs2CreER/+; Col4a5R471\* fl/y | + | 14-18w | 6 |

## Slide 3
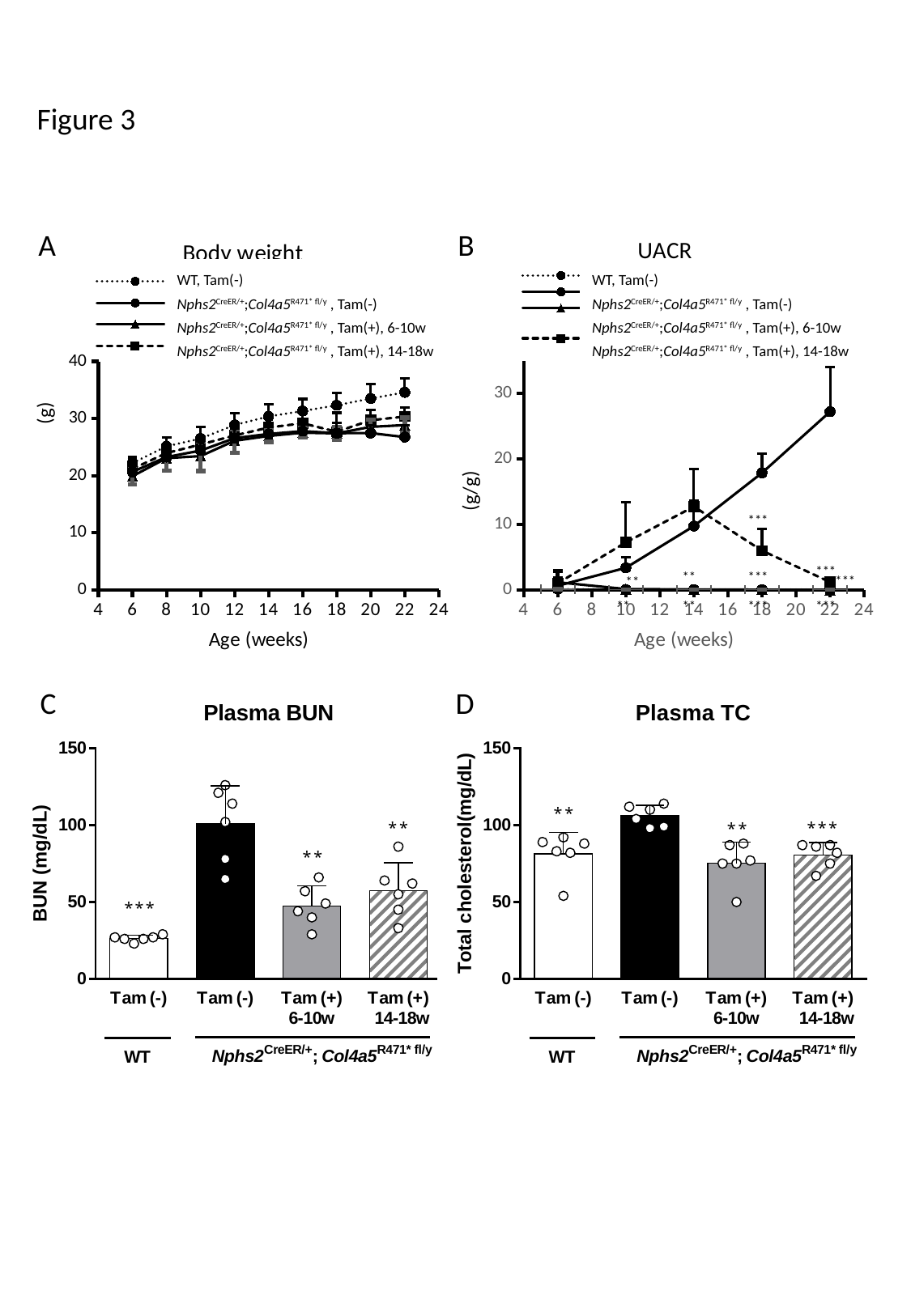

Figure 3
A
### Chart: Body weight
| Category | wild/wild, Tam(-) | flox/CreERT2, Tam(-) | flox/CreERT2, Tam(+), 6-10w | flox/CreERT2, Tam(+), 14-18w |
|---|---|---|---|---|B
### Chart: UACR
| Category | wild/wild, Tam(-) | flox/CreERT2, Tam(-) | flox/CreERT2, Tam(+), 6-10w | flox/CreERT2, Tam(+), 14-18w |
|---|---|---|---|---|WT, Tam(-)
Nphs2CreER/+;Col4a5R471* fl/y , Tam(-)
Nphs2CreER/+;Col4a5R471* fl/y , Tam(+), 6-10w
Nphs2CreER/+;Col4a5R471* fl/y , Tam(+), 14-18w
WT, Tam(-)
Nphs2CreER/+;Col4a5R471* fl/y , Tam(-)
Nphs2CreER/+;Col4a5R471* fl/y , Tam(+), 6-10w
Nphs2CreER/+;Col4a5R471* fl/y , Tam(+), 14-18w
***
***
**
***
***
**
**
**
***
***
C
D

## Slide 4
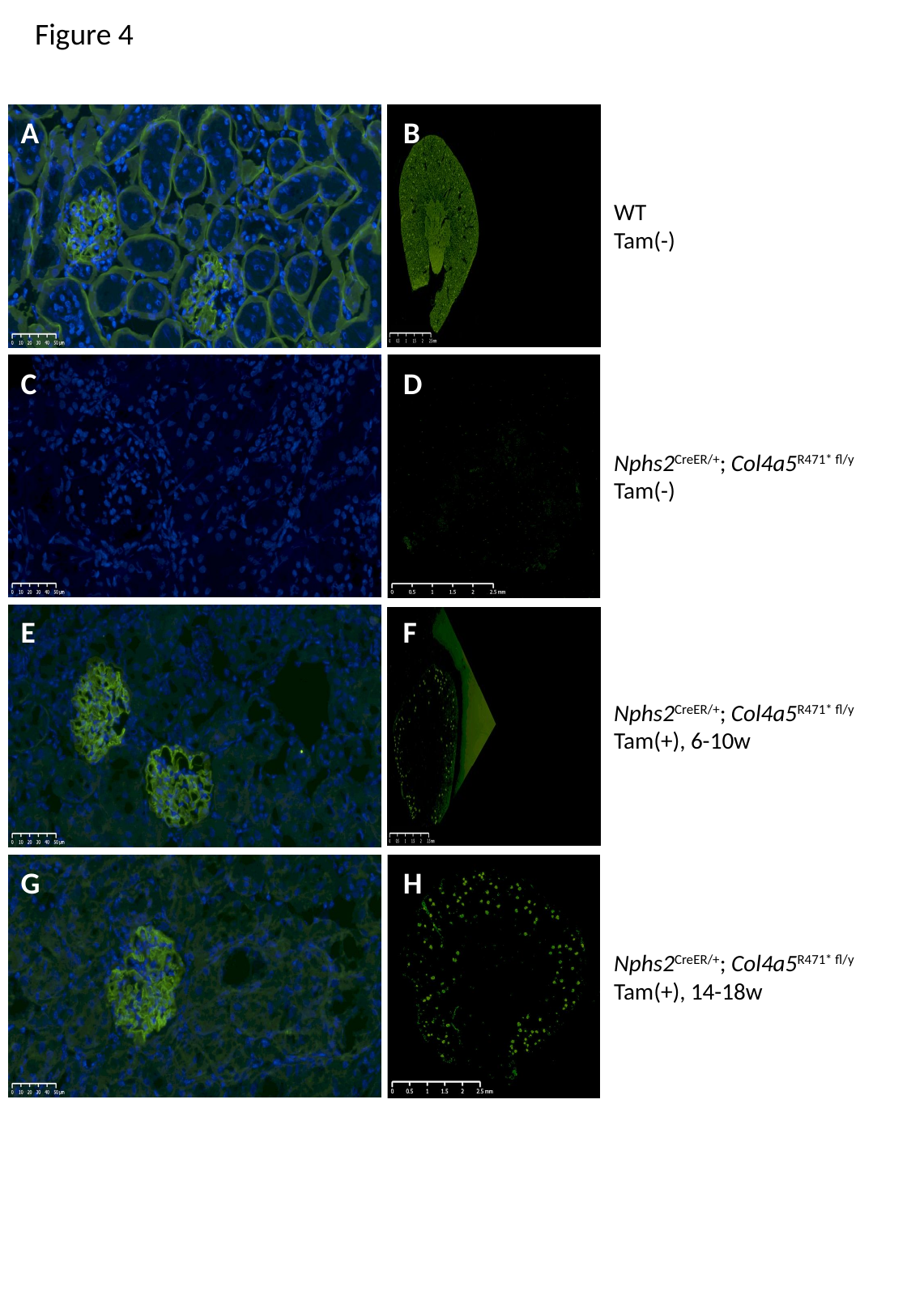

Figure 4
B
A
WT
Tam(-)
C
D
Nphs2CreER/+; Col4a5R471* fl/y
Tam(-)
E
F
Nphs2CreER/+; Col4a5R471* fl/y
Tam(+), 6-10w
G
H
Nphs2CreER/+; Col4a5R471* fl/y
Tam(+), 14-18w

## Slide 5
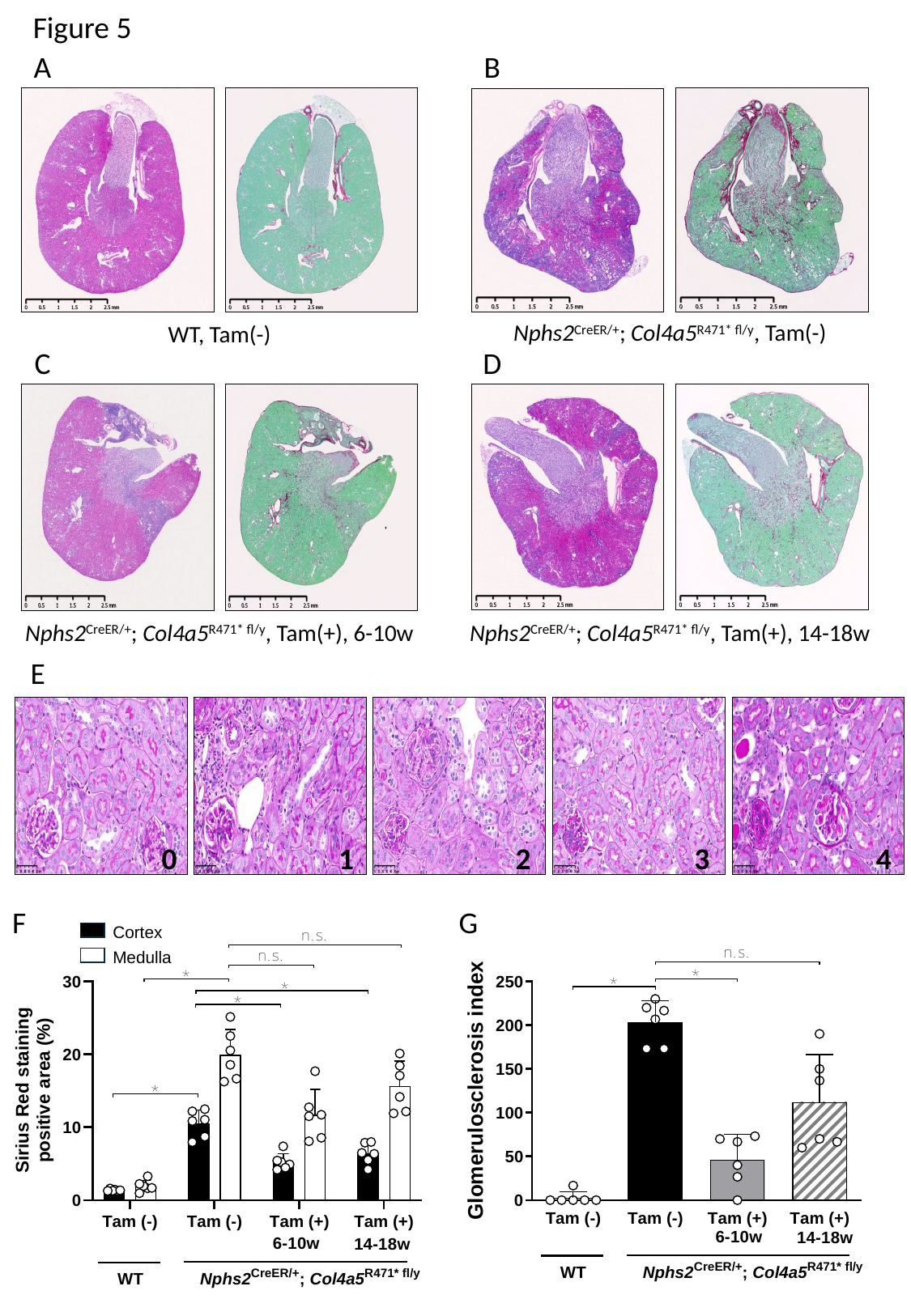

Figure 5
A
B
Nphs2CreER/+; Col4a5R471* fl/y, Tam(-)
WT, Tam(-)
C
D
Nphs2CreER/+; Col4a5R471* fl/y, Tam(+), 14-18w
Nphs2CreER/+; Col4a5R471* fl/y, Tam(+), 6-10w
E
0
1
2
3
4
F
G
Cortex
Medulla

## Slide 6
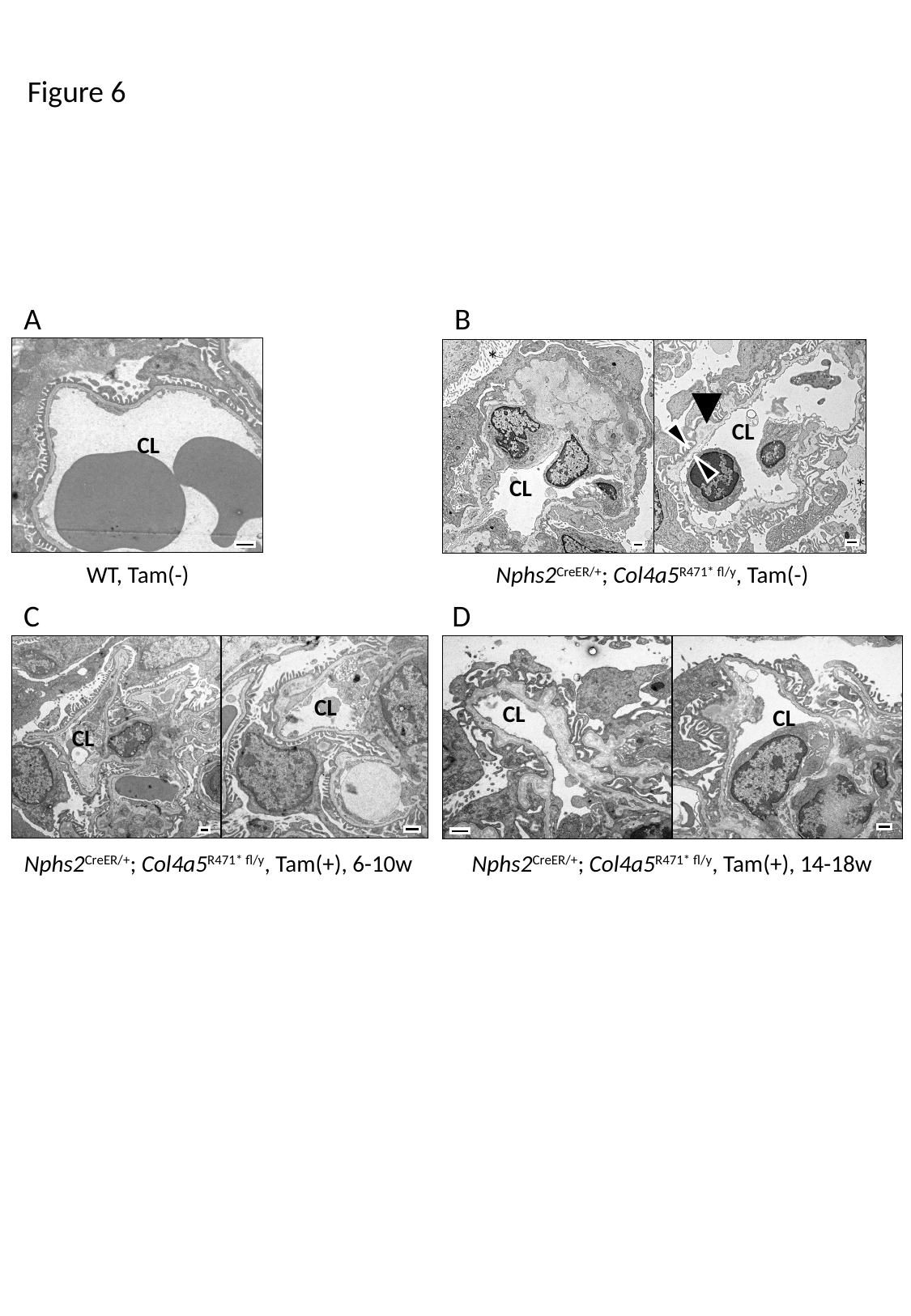

Figure 6
A
B
CL
*
CL
*
CL
WT, Tam(-)
Nphs2CreER/+; Col4a5R471* fl/y, Tam(-)
C
D
CL
CL
CL
CL
CL
Nphs2CreER/+; Col4a5R471* fl/y, Tam(+), 6-10w
Nphs2CreER/+; Col4a5R471* fl/y, Tam(+), 14-18w
